# Circulating basophil count as a prognostic marker of tumor aggressiveness and survival outcomes in colorectal cancer

**DOI:** 10.1186/s40169-019-0255-4

**Published:** 2020-02-10

**Authors:** Qi Liu, Dakui Luo, Sanjun Cai, Qingguo Li, Xinxiang Li

**Affiliations:** 1 Department of Colorectal Surgery, Fudan University Shanghai Cancer Center, #270 Dongan Road, Xuhui District, Shanghai, 200032 China; 2 Department of Oncology, Shanghai Medical College, Fudan University, Shanghai, China

**Keywords:** Basophils, Carcinoembryonic antigen, Classifier, Colorectal cancer, Immune/inflammation

## Abstract

**Background:**

Accumulating evidence demonstrated immune/inflammation-related implications of basophils in affecting tissue microenvironment that surrounded a tumor, and this study aimed to elucidate the clinical value of serum basophil count level.

**Methods:**

Between December 2007 and September 2013, 1029 patients diagnosed with stage I–III CRC in Fudan University Shanghai Cancer Center meeting the essential criteria were identified. The Kaplan–Meier method was used to construct the survival curves. Several Cox proportional hazard models were constructed to assess the prognostic factors. A simple predictor (CB classifier) was generated by combining serum basophil count and serum carcinoembryonic antigen (CEA) level which had long been accepted as the most important and reliable prognostic factor in CRC.

**Results:**

The preoperative basophils count < 0.025*10^9^/L was strongly associated with higher T stage, higher N stage, venous invasion, perineural invasion, elevated serum CEA level, and thus poor survival (P < 0.05). Moreover, multivariate Cox analysis showed that patients with low level of preoperative basophils count had an evidently poorer DFS [Hazard ratio (HR) = 2.197, 95% CI 1.868–2.585].

**Conclusions:**

As a common immune/inflammation-related biomarker available from the blood routine examination, low level of preoperative serum basophil count was associated with aggressive biology and indicated evidently poor survival. Preoperative serum basophil count would be a useful and simple marker for the management of CRC patients.

## Background

Colorectal cancer (CRC) is one of the most commonly diagnosed cancers among men and women [1]. The American Joint Committee on Cancer (AJCC) staging system has long been widely accepted for prognostic prediction and clinical management on the basis of the invasion extent of primary tumor (T stage), lymph node status (N stage), and distant spread (M stage).

However, the AJCC stage may fail to accurately distinguish patients at high risk of tumor recurrence and cancer death. In 2005, the AJCC once issued a request for proposals to develop staging methods based on other available information beyond the conventional tumor node metastases (TNM) staging [2, 3]. Recently, many new biomarkers, such as serum amyloid A (SAA), Ezrin protein, and mean corpuscular volume (MCV), have been reported to be associated with the prognosis of CRC [4]. However, reliable, convenient and low-cost biomarkers that can be used to optimally predict oncological outcomes, guide treatment decision, and can be put into a real clinical application are still lacking.

A number of previous studies demonstrated that the infiltration of inflammatory cells in colorectal tumors was associated with the recurrence and survival of patients with CRC [5, 6]. Available from the blood routine examination, basophils count is a common immune/inflammation-related biomarker. Accumulating evidence demonstrated immune/inflammation-related implications of basophils in affecting the tissue microenvironment that surrounded a tumor. [7–11]. In addition, serum carcinoembryonic antigen (CEA) had long been accepted as the most important and reliable prognostic factor despite the existence of several tumor markers and prognostic indicators in CRC [12]. This study aimed to elucidate the clinical value of serum basophil count level.

### Patients and methods

#### Patient selection from the Fudan University Shanghai Cancer Center (FUSCC) database

Between December 2007 and September 2013, patients diagnosed with stages I–III CRC in FUSCC meeting the following criteria were included in the analyses: (1) aged more than 18 years; (2) diagnosed with stages I–III CRC by histopathology, including adenocarcinoma, mucinous and signet ring cell carcinoma; (3) not receiving neoadjuvant therapy and laboratory blood tests performed before surgery; (4) underwent radical resection of primary tumor without positive surgical margin; (5) not receiving immunosuppressive therapy or receiving anti-inflammatory medicines; (6) without chronic inflammatory disease and (7) with complete relevant demographic and clinicopathologic data. In addition, for the sake of research, patients lost to follow-up within 5 years were excluded from the analyses.

The demographic and clinicopathological characteristics of patients were extracted from the FUSCC database. All patients were restaged according to the most recent of AJCC staging system (8th edition). 5-Fu-based adjuvant chemotherapy was recommended for both high-risk pathological stage II CRC and pathological stage III disease. Routine laboratory results including blood routine examination (neutrophil, lymphocyte, monocyte, eosinophil, basophil and platelet counts) and CEA level were extracted in retrospective medical records. All the blood samples were taken from patients within 3 days prior to the radical resection.

The main outcomes of interest used in this study were disease-free survival (DFS) and overall survival (OS). Overall survival was the time from diagnosis to patient death and DFS was calculated from the date of diagnosis to the date of the first event of recurrence, distant metastasis or death. This study was approved by the Ethical Committee and Institutional Review Board of FUSCC.

#### Selection of immune/inflammation-related biomarker in blood routine examination

Considering most of tumor recurrence or distant metastasis occurred within 5 years, 5-year DFS was then used as the outcome. For each immune/inflammation-related index (including neutrophil, lymphocyte, monocyte, eosinophil, basophil and platelet counts) in the blood routine examination, the sensitivity and specificity of each outcome, including tumor recurrence, distant metastasis, or death, were plotted, thus generating a receiver operating characteristic (ROC) curve. Next, the cutoff threshold using the Youden’s index and X-tile 3.6.1 software 20 (Yale University, New Haven, CT, USA) was selected to best dichotomize whether to be disease-free after 5 years. The cut-off score was also used to categorize all patients with CRC into high- and low-risk groups [13].

#### Statistical analyses

The clinicopathological characteristics of patients were compared according to the basophils count level using the Pearson’s Chi squared test. The Kaplan–Meier method was used to construct the survival curves in the present study, and the univariate survival difference was determined using the log-rank test. Several Cox proportional hazard models were constructed to assess the prognostic factors. Only the clinicopathological characteristics that showed prognostic significance (log-rank, P < 0.20) in the univariate Cox analyses were included in the multivariate analyses. Two-sided *P *< 0.05 was considered statistically significant. Data were statistically analyzed using the SPSS version 22 (IBM Corporation, Armonk, NY, USA).

## Results

### Basophils count level was a good immune/inflammation-related biomarker to predict 5-year DFS

As shown in Additional file 1: Figure S1, the discrimination ability of seven immune/inflammation-related markers was compared by area under the ROC for 5-year DFS. Of these, the Area Under Curve (AUC) of basophils count was 0.716 [95% confidence interval (CI) 0.684–0.747], which was the strongest one for predicting 5-year DFS in patients diagnosed with CRC. The ROC curve for the basophils count clearly illustrated the point on the curve closest to (0.0, 1.0), which maximized both sensitivity (0.562) and specificity (0.777) for the outcome. Also, we used X-tile program to determine the optimal cutoff value for basophils count, and the result was the same as the Youden’s index (Additional file 2: Figure S2). Then, the cutoff score 0.025*10^9^/L was used to categorize all patients with CRC into two groups, basophils count less than 0.025*10^9^/L as the high-risk group (B−) and count more than 0.025*10^9^/L as the low-risk one (B+).

### Patient characteristics of the whole cohort

In total, 1029 patients diagnosed with stage I–III CRC between December 2007 and September 2013 with more than 5 years of follow-up time were identified. The median follow-up time among censored patients for DFS and OS was 70 and 63 months, respectively. Of all the patients included in the study, 607 (59.0%) were men and 422 (41.0%) were women. The median age [interquartile range (IQR)] was 59 (IQR 52–67) years; 414 (40.2%) patients were confirmed with colon cancer and 615 (59.8%) with rectal cancer.

The baseline characteristics in high- and low-risk groups according to the basophils count level were summarized in Additional file 4: Table S1. It was easy to find that B− was more likely to be associated with higher T stage, higher N stage, venous invasion, perineural invasion and elevated serum CEA level (CEA+, serum CEA more than 5.2 ng/mL was considered to be elevated in FUSCC), meaning that the low level of basophils count was a surrogate for biologically aggressive disease (P < 0.05).

### Serum basophil count level was strongly associated with DFS and OS of CRC

As for the basophils count, B− presented a significantly decreased 5-year OS rate (49.2% vs. 72.2%, P = 0.02); the DFS difference was even more pronounced and the 5-year DFS rates of B− and B+ were 20.4% and 53.3%, respectively (P < 0.001, Additional file 3: Fig. S3).

Multivariate Cox analysis showed that high level of basophil count was an independent prognostic factor of DFS [Hazard ratio (HR) = 2.197, 95% CI 1.868–2.585, P < 0.001; Table 1]. And low level of basophil count was independently associated with 74.8% increased risk of overall mortality (HR = 1.748, 95% CI 1.410–2.168, P < 0.001; Table 2).

**Table 1:** Univariate and multivariate Cox analyses of DFS in the whole cohort

Variable	University analysis		Multivariate analysis	
	HR (95% CI)	*P*	HR (95% CI)	*P*
CEA level		< 0.001		< 0.001
Normal	Reference		Reference	
Elevated	2.439 (2.088–2.849)		1.879 (1.598–2.211)	
Basophil level		< 0.001		< 0.001
≥ 0.025*10^9^/L	Reference		Reference	
< 0.025*10^9^/L	2.381 (2.036–2.784)		2.197 (1.868–2.585)	
T stage		< 0.001		0.025
T1	Reference		Reference	
T2	1.681 (0.907–3.115)	0.099	1.331 (0.713–2.485)	0.369
T3	2.854 (1.583–5.147)	< 0.001	1.727 (0.942–3.168)	0.077
T4a	4.158 (2.341–7.387)	< 0.001	1.972 (1.082–3.595)	0.027
T4b	5.164 (2.650–10.062)	< 0.001	2.058 (1.030–4.112)	0.041
N stage		< 0.001		< 0.001
N0	Reference		Reference	
N1a	1.511 (1.118–2.042)	0.007	1.236 (0.902–1.693)	0.188
N1b	2.099 (1.619–2.722)	< 0.001	1.907 (1.447–2.513)	< 0.001
N1c	2.705 (2.122–3.446)	< 0.001	1.798 (1.385–2.334)	< 0.001
N2a	2.699 (2.125–3.430)	< 0.001	1.848 (1.403–2.434)	< 0.001
N2b	3.508 (2.778–4.430)	< 0.001	2.685 (2.040–3.535)	< 0.001
Gender		0.382		
Male	Reference			
Female	0.932 (0.797–1.091)			
Histology		0.340		
Adenocarcinoma	Reference			
Mucinous adenocarcinoma	1.023 (0.832–1.257)	0.832		
Signet ring cell carcinoma	2.010 (1.255–3.218)	0.004		
Tumor location		0.431		
Right colon	Reference			
Left colon	0.847 (0.658–1.089)	0.195		
Rectum	0.935 (0.778–1.123)	0.470		
Tumor grade		< 0.001		0.128
Well	Reference		Reference	
Moderate	1.610 (1.090–2.378)		1.441 (0.971–2.140)	0.070
Poor	2.287 (1.533–3.412)		1.523 (1.014–2.288)	0.043
Venous invasion		< 0.001		0.732
No	Reference		Reference	
Yes	1.679 (1.434–1.965)		1.032 (0.862–1.235)	
Perineural invasion		< 0.001		0.001
No	Reference		Reference	
Yes	1.952 (1.663–2.291)		1.357 (1.136–1.620)	
Lymph nodes dissected in total		0.048		0.004
< 12	Reference		Reference	
≥ 12	1.222 (1.002–1.491)		0.735 (0.596–0.905)	
Age (years)		< 0.001		< 0.001
< 65	Reference		Reference	
≥ 65	1.483 (1.264–1.740)		1.509 (1.277–1.783)	
Adjuvant chemotherapy		0.045		0.294
No	Reference		Reference	
Yes	1.242 (1.005–1.534)		0.884 (0.702–1.113)

**Table 2:** Univariate and multivariate Cox analyses of OS in the whole cohort

Variable	University analysis		Multivariate analysis	
	HR (95% CI)	*P*	HR (95% CI)	*P*
CEA level		< 0.001		< 0.001
Normal	Reference		Reference	
Elevated	3.414 (2.754–4.231)		2.417 (1.928–3.030)	
Basophil level		< 0.001		< 0.001
≥ 0.025*10^9^/L	Reference		Reference	
< 0.025*10^9^/L	2.150 (1.746–2.648)		1.748 (1.410–2.168)	
T stage		< 0.001		0.038
T1	Reference		Reference	
T2	0.882 (0.375–2.074)	0.773	0.553 (0.232–1.318)	0.181
T3	2.201 (1.013–4.784)	0.046	0.945 (0.419–2.129)	0.891
T4a	3.638 (1.716–7.717)	0.001	1.136 (0.510–2.531)	0.755
T4b	6.344 (2.731–14.736)	< 0.001	1.312 (0.532–3.232)	0.555
N stage		< 0.001		<0.001
N0	Reference		Reference	
N1a	1.744 (1.120–2.717)	0.014	1.610 (1.008–2.574)	0.046
N1b	2.094 (1.407–3.116)	< 0.001	2.124 (1.384–3.259)	0.001
N1c	3.626 (2.584–5.089)	< 0.001	2.634 (1.811–3.830)	<0.001
N2a	3.995 (2.883–5.536)	< 0.001	2.764 (1.891–4.041)	<0.001
N2b	6.292 (4.609–8.591)	< 0.001	4.590 (3.150–6.687)	<0.001
Gender		0.351		
Male	Reference			
Female	1.104 (0.897–1.358)			
Histology		< 0.001		0.447
Adenocarcinoma	Reference		Reference	
Mucinous adenocarcinoma	1.292 (0.996–1.676)	0.054	1.125 (0.833–1.519)	0.443
Signet ring cell carcinoma	3.166 (1.881–5.330)	< 0.001	1.402 (0.788–2.493)	0.250
Tumor location		< 0.001		0.005
Right colon	Reference		Reference	
Left colon	0.555 (0.399–0.774)	0.001	0.590 (0.420–0.829)	0.002
Rectum	0.632 (0.503–0.795)	< 0.001	0.742 (0.584–0.944)	0.015
Tumor grade		< 0.001		0.119
Well	Reference		Reference	
Moderate	2.123 (1.126–4.004)	0.020	1.874 (0.986–3.564)	0.055
Poor	3.590 (1.890–6.819)	< 0.001	2.013 (1.036–3.910)	0.039
Venous invasion		< 0.001		0.033
No	Reference		Reference	
Yes	2.165 (1.762–2.660)		1.290 (1.021–1.630)	
Perineural invasion		< 0.001		0.164
No	Reference		Reference	
Yes	1.867 (1.509–2.310)		1.181 (0.934–1.493)	
Lymph nodes dissected in total		0.325		
< 12	Reference			
≥ 12	1.140 (0.878–1.480)			
Age (years)		< 0.001		< 0.001
< 65	Reference		Reference	
≥ 65	1.849 (1.501–2.276)		1.658 (1.329–2.068)	
Adjuvant chemotherapy		0.111		< 0.001
No	Reference		Reference	
Yes	0.813 (0.631–1.049)		0.491 (0.369–0.652)	

**Fig. 1:**
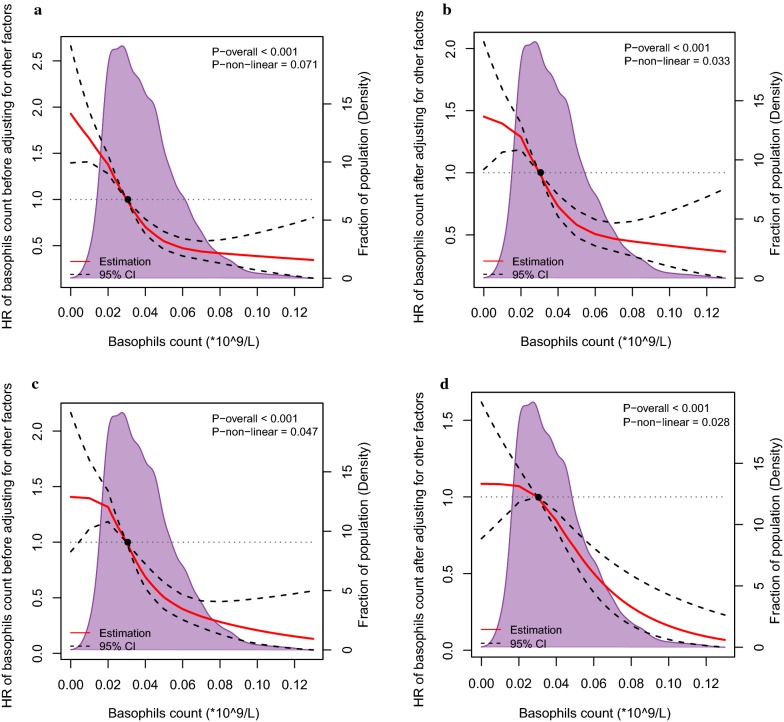
Preoperative serum basophil count and the corresponding hazard ratios on a continuous scale, including (**a**). DFS before adjusting for other prognostic factors; (**b**). DFS after adjusting for other prognostic factors; (**c**). OS before adjusting for other prognostic factors; (**d**). OS after adjusting for other prognostic factors. Analyses were conducted using restricted cubic splines, with hazard ratios and 95% confidence intervals from univariate to multivariate Cox proportional hazards regression. The basophils count of 0.03*10^9^/L was chosen as the reference. The purple area indicated the distribution of concentration of the basophils count

Using restricted cubic splines, in addition, we also showed the preoperative serum basophil count and the corresponding hazard ratios on a continuous scale (Fig. 1a–d). It was evident that basophils count was negatively correlated with the mortality and morbidity, both before and after adjusting for other prognostic factors.

While CEA had long been accepted to be the most important serum tumor biomarker of CRC, the results of multivariate analyses indicated that the prognostic prediction ability of serum basophil level was not be covered up by CEA level and other clinicopathological factors, in both OS and DFS, meaning that the two serum variables could be grouped for a better clinical application with the aforementioned definition of basophils count < 0.025*10^9^/L as B−, basophils count ≥ 0.025*10^9^/L as B+, elevated serum CEA (> 5.2 ng/mL) as CEA + , and normal serum CEA (≤ 5.2 ng/mL) as CEA−.

We then constructed a new variable called CB classifier, consisting of CEA−, B−; CEA−, B+; CEA+, B− and CEA+, B+. Considering univariate Cox analyses of DFS showed that HR of CEA+, B+ (HR = 1.033, 95% CI  0.831–1.285) was not statistically different from HR of CEA−, B−, CB classifier of DFS was redefined as CEA−, B−/CEA+, B+; CEA−, B+ and CEA+, B−. CB classifier of OS was still CEA−, B−; CEA−, B+; CEA+, B− and CEA+, B+.

**Fig. 2:**
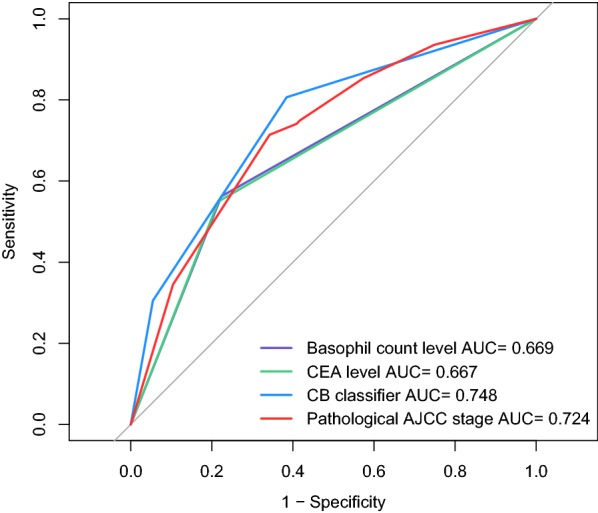
Receiver operating characteristic curves for serum basophil count level, CEA level, CB classifier and pathological AJCC stage

The ROC curves of DFS for basophils count level, CEA level, CB classifier and pathological AJCC TNM staging system (stages I–IIIC) were plotted. The AUC for the CB classifier (0.748) was significantly greater than the AUC for basophils count level, CEA level, and pathological AJCC TNM staging system (Fig. 2).

### Prognosis of serum basophil count level after the combination with AJCC TNM staging system and the interesting phenomenon of stage migration

**Fig. 3:**
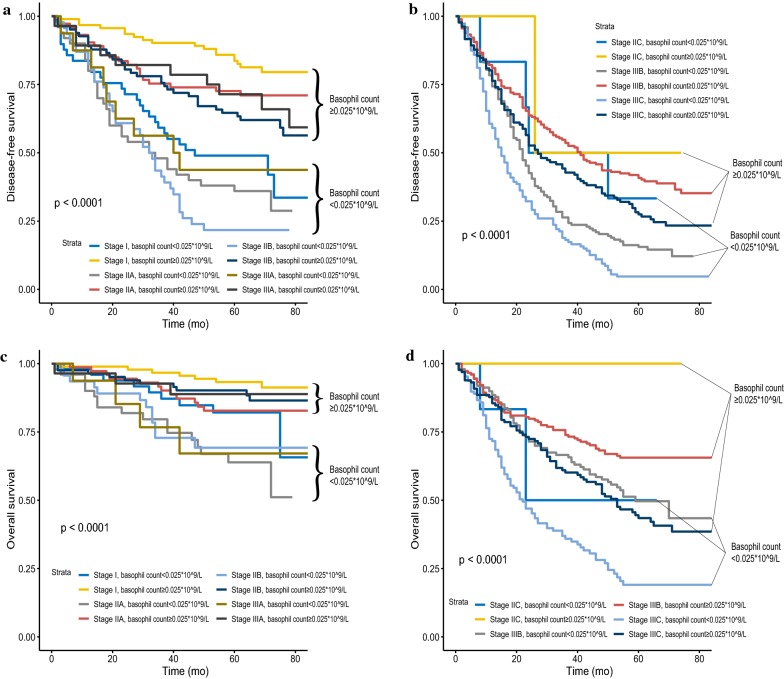
Kaplan–Meier survival curves of the serum basophil count level in respective AJCC TNM staging system, including DFS of (**a**). stage I, stage IIA, stage IIB and stage IIIA (**b**). stage IIC, stage IIIB and stage IIIC; and OS of (**c**). stage I, stage IIA, stage IIB and stage IIIA (**d**). stage IIC, stage IIIB and stage IIIC

Survival curves of B− and B+ after combining with the AJCC TNM staging system were plotted (Fig. 3). As shown in Fig. 3a, b, the B− patients showed a statistically significant decrease in DFS compared with the B+ patients (*P* < 0.05) in the respective AJCC TNM stages except in stage IIC (P = 0.611) and stage IIIA (P = 0.094), which could be due to the small sample size of stage IIC (n = 8) and stage IIIA (n = 44) disease, while sample sizes of other stages were more than 123 (Table 3). OS was the same, the B− patients showed a statistically significant decrease in OS compared with the B− patients (*P* < 0.05) in the respective AJCC TNM stages except in stage IIC (P = 0.258) and stage IIIA (P = 0.128), which could be due to the relatively small sample size of stage IIC and stage IIIA disease (Fig. 3c, d).

**Table 3:** DFS of colorectal cancer after incorporation of serum basophil count level into conventional AJCC TNM staging system

AJCC TNM staging system				After incorporation of C-stage into AJCC stage			
Stage	No. of patients	Disease-free survival		Stage	No. of patients	Disease-free survival	
		HR (95% CI)	P value			HR (95% CI)	P value
I	141	1	–	I B−	49	1	–
				I B+	92	0.270 (0.149–0.488)	< 0.001
IIA	123	1.340 (0.895–2.005)	0.155	IIA B−	50	1.174 (0.701–1.968)	0.542
				IIA B+	73	0.407 (0.228–0.727)	0.002
IIB	128	1.668 (1.138–2.447)	0.009	IIB B−	46	1.412 (0.850–2.345)	0.183
				IIB B+	82	0.548 (0.327–0.918)	0.022
IIC	8	1.501 (0.591–3.814)	0.393	IIC B−	6	1.238 (0.430–3.569)	0.692
				IIC B+	2	0.445 (0.060–3.318)	0.430
IIIA	44	1.317 (0.762–2.275)	0.324	IIIA B−	16	0.891 (0.414–1.919)	0.769
				IIIA B+	28	0.542 (0.260–1.133)	0.103
IIIB	327	2.617 (1.852–3.697)	< 0.001	IIIB B−	148	1.932 (1.249–2.988)	0.003
				IIIB B+	179	1.002 (0.643–1.559)	0.995
IIIC	258	3.478 (2.424–4.990)	< 0.001	IIIC B−	127	2.679 (1.714–4.187)	< 0.001
				IIIC B+	131	1.275 (0.809–2.008)	0.295

Moreover, the B− patients showed worse DFS in some stages compared with the B+ patients with higher AJCC stages (Fig. 3a, b). In stages I, IIA, IIB, and IIIA, one noteworthy finding was that B+, irrespective of the stage, was associated with better DFS compared with B− in all these stages; the B+ patients in stage IIB had better DFS compared with the B− patients in stage I (P = 0.037, Fig. 3a). Cox analyses were used to validate the interesting phenomenon of stage migration (Table 3). Consistent with the Kaplan–Meier survival curves, B− presented increased HRs compared with B+ in the respective AJCC stages and HRs of stage IIIC B+ (HR = 1.275; 95% CI 0.809–2.008, P = 0.295, using stage I B− as the reference) and stage IIIB B+ (HR = 1.002; 95% CI 0.643–1.559, P = 0.995, using stage I B− as the reference) did not even achieve statistical significance compared with stage I B−. The results of OS were similar to the results of DFS (Fig. 3c, d and Additional file 4: Table S2).

## Discussion

Although serum CEA had long been accepted as the most important and reliable prognostic factor in CRC, it had been reported that 20–30% of patients with CRC failed to produce elevated serum levels, which was considered as one of the major limitations in monitoring [12, 14]. Therefore, other biomarkers for the follow-up of those patients were necessary. The present study revealed that the preoperative serum basophil count < 0.025*10^9^/L was strongly associated with higher T stage, higher N stage, venous invasion, perineural invasion, elevated serum CEA level, and thus poor survival, irrespective of the AJCC stages (stage I–III). In addition, the stage migration of combination with serum basophil count level and conventional AJCC staging system (stage I–IIIC) also indicated the good prognostic prediction ability of preoperative serum basophil count level. The preoperative serum CB classifier even had a greater AUC for predicting 5-year DFS compared with the postoperative pathological AJCC TNM stage (stages I–IIIC) in patients diagnosed with CRC.

Discovered by Paul Ehrlich with the name of “Mastzellen”, the work on basophils began at the end of the 19th century [15]. Developing from hematopoietic stem cells, basophils typically become mature in the bone marrow, and then enter the bloodstream [16]. Normally, basophils are present in very low numbers in the circulation. Under certain circumstances, however, basophils can be activated, increase in number and move from the bloodstream to sites of infection and inflammation [17, 18]. However, for a long time, basophils did not get the intensive attention of investigators, not only owning to their sparse distribution (basophil only represented less than 1% of blood leukocytes) and short life span of about only 2–3 days, but also because of difficulty in exploring the in vivo function of basophils due to the lack of basophil-deficient mouse models [7, 16]. Recently, both basophil-depleting antibodies and basophil-deficient mouse models have been established, and then new roles for basophils have been identified, such as allergic reactions, regulation of acquired immunity and so on [8].

**Fig. 4:**
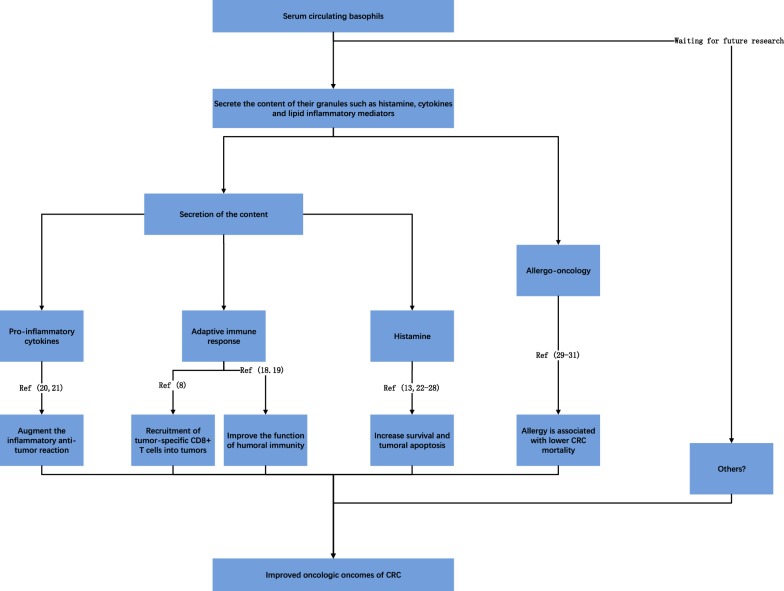
Schematic representation of the reasons for better oncologic outcome of CRC patients with high-level basophils count

Basophils have also been reported to participate in the regulation of tissues transforming into neoplasia. The tissue microenvironment that surrounds a tumor plays an important role in affecting the growth of cancer cells [19]. Furthermore, the accumulation of basophils in the tumor microenvironment has been demonstrated in both mouse models of cancer and primary human tumors, basophils are an important part of inflammatory cells in the tumor microenvironment [7, 9]. Basophils secrete the content of their granules such as histamine, cytokines and lipid inflammatory mediators, which polarize the immune reaction response for the immunoglobulin E (IgE)-dependent inflammatory and allergic reactions. The known reasons accounting for the association of high-level basophils count and better oncologic outcome of CRC were summarized in the following text (Fig. 4).

First of all, in 2016, Sektioglu et al. reported that basophilia in tumors was beneficial for T cell infiltration and tumor rejection. This study also showed that basophils had a critical role in tumor rejection and promoting the recruitment of tumor-specific CD8+ T cells into tumors, which could be exploited for future therapeutic interventions [8]. Second, basophils were also found to improve the function of humoral immunity. Basophils could secret a number of B-cell-modulating molecules, suggesting the participation of basophils in the regulation of humoral immunity. Activated basophils were shown to express CD40L, IL-4 and IL-6 to support B-cell proliferation and IgM and IgG1 production [20]. In 2010, Rodriguez Gomez et al. demonstrated that basophils could also supported plasma cell survival, both in vitro and in vivo [21].

In addition, it was also reported that basophils could release pro-inflammatory cytokines such as TNF-α, IL-6 and IL-1β, which augmented the inflammatory anti-tumor reaction, resulting in direct anti-neoplastic functions and inducing the apoptosis of tumor cells [22, 23]. Normally, basophilic granulocytes contained histamine at a much higher concentration compared with any other element in the blood element. Moreover, despite its scarcity, histamine in basophils accounted for half of the histamine in the normal bloodstream [15, 24]. As an endogenous biogenic amine widely distributed throughout the body, histamine [2-(4-imidazolyl)-ethylamine] is involved in numerous physio-pathological conditions. The four histamine receptor subtypes are widely distributed throughout the digestive system and all histamine receptor subtypes are involved in cancer, including CRC. Histamine could suppress colorectal tumorigenesis and severity of inflammation-associated colon cancer in HDC KO mice [25, 26]. Yet a low expression level of histamine receptors and histamine receptor gene was demonstrated in colon adenocarcinoma compared with normal colonic tissue [27]. Increased H2R gene expression was reported to be associated with improved prognosis in patients with CRC [28]. More importantly, histamine significantly increased median survival and tumoral apoptosis and histamine deficiency could promote inflammation-associated carcinogenesis, suggesting that histamine could also partly explain the favorable prognosis of patients with CRC having a high serum basophil count [29, 30].

Last but not least, allergo-oncology, officially ideated in 2006, aimed at studying the significance of IgE-mediated immune responses against tumors [31]. Several cancers, such as glioma, pancreatic cancer and pediatric leukemia have been reported to be inversely associated with allergy, [32]. Also, data collected from prospective studies, based on the patient-reported family history of allergy, demonstrated the presence of allergic diseases and lower CRC mortality [33]. In addition, from what had been discussed above, it was found that circulating IgE levels was very important in the anti-tumor activities of basophils. However, this information was not available since it was a retrospective one, and we hope future studies should include the circulating IgE levels into their analyses.

Additional unknown mechanisms might also contribute to the favorable prognosis of patients with CRC having a high-level serum basophil count. The findings of the present study could elicit further research on these underlying mechanisms. Two previous study investigated the relationship between basophils count level and prognosis of CRC, with just a cursory summary that a high level of pretreatment circulating basophils count correlated with a favorable prognosis of patients with CRC, which was in line with the findings of the present study [11, 34]. In fact, the two studies with small sample size did not focus on the clinical value of circulating basophil count, without comparison to the prognostic value of serum CEA which had long been accepted as the most important and reliable prognostic factor in CRC, without detailed descriptions of the prognostic significance of circulating basophil count or comprehensive investigation of the mechanisms behind. In addition, a low level of basophils count correlated with an increased number of lung metastases was also found in breast cancer [10]. However, the presence of basophils in tumor-draining lymph nodes and tumors correlates with Th2 inflammation and reduced survival in pancreatic cancer [35]. Basophilia (> 250/μL) was reported to reduce the survival of myelodysplastic syndromes (MDS) [36]. Besides, two previous studies found no prognostic value of pretreatment basophils count in the prognosis of oral squamous cell carcinoma and breast cancer [37, 38]. The difference in the prognostic roles reported in the four recent studies and the present study might be due to the different types of cancers, the sample sizes and more importantly, the different circulating IgE levels Different circulating IgE levels of patients might greatly affect anti-tumor activities of basophils, however, this information was not mentioned in the two previous studies.

As a long-accepted prognostic biomarker in CRC, the elevated serum CEA level (> 5.2 ng/mL) was also significantly associated with a poorer prognosis in the present study. Above all, the prognostic value of basophils count was not covered up by CEA, resulting in a novel prognostic index named “CB classifier” which had a great AUC for predicting 5-year DFS. The findings of the present study, if validated in other large population-based studies, would provide us better understanding of tumor biology and be of great significance.

This study had some limitations. First, it was a retrospective and single-center study. Despite being a large population-based study with a long follow-up, its sample size still needed to be maximize in a multicenter one. Therefore, a large prospective multicenter study focused on the clinical value of preoperative serum basophil count was necessary to validate the present findings. Second, circulating IgE levels, which might correlate with the anti-tumor effect of basophils, were not available in this retrospective study. Finally, this study took a relatively long period between 2007 and 2013, which might introduce some historical biases on treatment strategy.

## Conclusion

In summary, as a common immune/inflammation-related biomarker available from the blood routine examination, basophils count negatively correlated with the mortality and morbidity of CRC patients. Low level of preoperative basophils count was associated with aggressive biology and indicated evidently poor survival. Preoperative serum basophil count, whose prognostic value was not covered up by serum CEA level, would be a useful and simple marker for the management of CRC patients.

## Supplementary information

**Additional file 1: Figure S1.** Receiver operating characteristic curves for immune/inflammation-relatedbiomarkers in blood routine examination, including basophil, white blood cell (WBC), platelet, neutrophil, lymphocyte, monocyte and eosinophil.

**Additional file 2: Figure S2.** X-tile analyses of DFS were performed to determine the optimal cut-off valuesfor basophils count.

**Additional file 3: Figure S3.** Kaplan–Meier (a). DFS and (b). OS curves of the serum basophil count level.

**Additional file 4: Table S1.** Comparison of baseline characteristics of the whole cohort by the serum basophil level. **Table S2.** OS of colorectal cancer after incorporation of serum basophil count level into conventional AJCC TNM staging system.

## Data Availability

The data used in the current study are available from the corresponding author on reasonable request.

## References

[1] Siegel RL, Miller KD, Jemal A (2019). Cancer statistics. CA Cancer J Clin.

[2] Park JH, Watt DG, Roxburgh CS (2016). Colorectal cancer, systemic inflammation, and outcome: staging the tumor and staging the host. Ann Surg.

[3] Weiser MR, Mithat GN, Chou JF (2011). Predicting survival after curative colectomy for cancer: individualizing colon cancer staging. J Clin Oncol Off J Am Soc Clin Oncol.

[4] Marks KM, West NP, Morris E, Quirke P (2018). Clinicopathological, genomic and immunological factors in colorectal cancer prognosis. Br J Surg.

[5] Roxburgh CS, McMillan DC (2012). The role of the in situ local inflammatory response in predicting recurrence and survival in patients with primary operable colorectal cancer. Cancer Treat Rev.

[6] Chan JC, Chan DL, Diakos CI (2016). The lymphocyte-to-monocyte ratio is a superior predictor of overall survival in comparison to established biomarkers of resectable colorectal cancer. Ann Surg.

[7] Rigoni A, Colombo MP, Pucillo C (2018). Mast cells, basophils and eosinophils: from allergy to cancer. Semin Immunol.

[8] Sektioglu IM, Carretero R, Bulbuc N (2016). Basophils promote tumor rejection via chemotaxis and infiltration of CD8+ T cells. Cancer Res.

[9] Sonia M, Elena B, Alice Amaranta C (2015). Mast cells, basophils and B cell connection network. Mol Immunol.

[10] Wang C, Chen YG, Gao JL (2015). Low local blood perfusion, high white blood cell and high platelet count are associated with primary tumor growth and lung metastasis in a 4T1 mouse breast cancer metastasis model. Oncol Lett.

[11] Wei Y, Zhang X, Wang G (2018). The impacts of pretreatment circulating eosinophils and basophils on prognosis of stage I-III colorectal cancer. Asia Pac J Clin Oncol.

[12] Haglund C (2007). Tumour markers in colorectal cancer: European group on tumour markers (EGTM) guidelines for clinical use. Eur J Cancer.

[13] Camp RL, Marisa DF, Rimm DL (2004). X-tile: a new bio-informatics tool for biomarker assessment and outcome-based cut-point optimization. Clin Cancer Res.

[14] Duffy MJ, van Dalen A, Haglund C (2003). Clinical utility of biochemical markers in colorectal cancer: European group on tumour markers (EGTM) guidelines. Eur J Cancer.

[15] Enrico C, Beatrice N, Domenico R (2011). The history of the controversial relationship between mast cells and basophils. Immunol Lett.

[16] Arinobu Y, Iwasaki H, Akashi K (2009). Origin of basophils and mast cells. Allergology.

[17] Chirumbolo S (2012). State-of-the-art review about basophil research in immunology and allergy: is the time right to treat these cells with the respect they deserve?. Blood Transfus.

[18] Yamanishi Y, Miyake K, Iki M (2017). Recent advances in understanding basophil-mediated Th2 immune responses. Immunol Rev.

[19] Kempen LC, Ruiter DJ, Muijen GN, Coussens LM (2004). The tumor microenvironment: a critical determinant of neoplastic evolution. Eur J Cell Biol.

[20] Denzel A, Maus UA, Rodriguez Gomez M (2008). Basophils enhance immunological memory responses. Nat Immunol.

[21] Manuel RG, Yvonne T, Nicole G (2010). Basophils support the survival of plasma cells in mice. J Immunol.

[22] Gooch JL, Lee AV, Yee D (1998). Interleukin 4 inhibits growth and induces apoptosis in human breast cancer cells. Cancer Res.

[23] Gordon JR, Galli SJ (1990). Mast cells as a source of both preformed and immunologically inducible TNF-alpha/cachectin. Nature.

[24] Graham HT, Lowry OH, Wheelwright F (1955). Distribution of histamine among leukocytes and platelets. Blood.

[25] Kennedy L, Hodges K, Meng F (2012). Histamine and histamine receptor regulation of gastrointestinal cancers. Transl Gastrointest Cancer.

[26] Massari NA, Nicoud MB, Medina VA (2018). Histamine receptors and cancer pharmacology: an update. Br J Pharmaco.

[27] Boer K, Helinger E, Helinger A (2008). Decreased expression of histamine H1 and H4 receptors suggests disturbance of local regulation in human colorectal tumours by histamine. Eur J Cell Biol.

[28] Gao C, Ganesh BP, Shi Z (2017). Gut Microbe-mediated suppression of inflammation-associated colon carcinogenesis by luminal histamine production. Am J Pathol.

[29] Lamas DJ, Martinel C, Maximo C, Eliana C (2013). Therapeutic potential of histamine H4 receptor agonists in triple-negative human breast cancer experimental model. Br J Pharmacol.

[30] Yang XD, Ai W, Asfaha S (2011). Histamine deficiency promotes inflammation-associated carcinogenesis through reduced myeloid maturation and accumulation of CD11b+ Ly6G+ immature myeloid cells. Nat Med.

[31] Jensen-Jarolim E, Untersmayr E, Knittelfelder R et al (2006) Allergo-Oncology: the role of IgE in tumor defense. In 26th Symposium of the Collegium Internationale Allergologicum. pp. 5–10

[32] Turner MC, Yue C, Daniel K, Parviz G (2010). An overview of the association between allergy and cancer. Int J Cancer.

[33] Jacobs EJ, Gapstur SM, Newton CC (2013). Hay Fever and asthma as markers of atopic immune response and risk of colorectal cancer in three large cohort studies. Cancer Epidemiol Biomarkers Prev.

[34] Wu J, Ge XX, Zhu W (2019). Values of applying white blood cell counts in the prognostic evaluation of resectable colorectal cancer. Mol Med Rep.

[35] De ML, Woermann S, Brunetto E (2016). Basophil recruitment into tumor draining lymph nodes correlates with Th2 inflammation and reduced survival in pancreatic cancer patients. Cancer Res.

[36] Wimazal F, Germing U, Kundi M (2010). Evaluation of the prognostic significance of eosinophilia and basophilia in a larger cohort of patients with myelodysplastic syndromes. Cancer.

[37] Yasemin Benderli C, Alaettin A, Mehmet Faik C, Hasan M (2014). Lack of prognostic value of blood parameters in patients receiving adjuvant radiotherapy for breast cancer. Asian Pac J Cancer Prev.

[38] Grimm M, Rieth J, Hoefert S (2016). Standardized pretreatment inflammatory laboratory markers and calculated ratios in patients with oral squamous cell carcinoma. Eur Arch Otorhinolaryngol.

