# Supplementary material for: Circulating basophil count as a prognostic marker of tumor aggressiveness and survival outcomes in colorectal cancer
Source: Clin Transl Med. 2020 Feb 10;9:6. doi: 10.1186/s40169-019-0255-4 (PMC7008108; doi:10.1186/s40169-019-0255-4)

Figure S1. Receiver operating characteristic curves for immune/inflammation-related biomarkers in blood routine examination, including basophil, white blood cell (WBC), platelet, neutrophil, lymphocyte, monocyte and eosinophil.

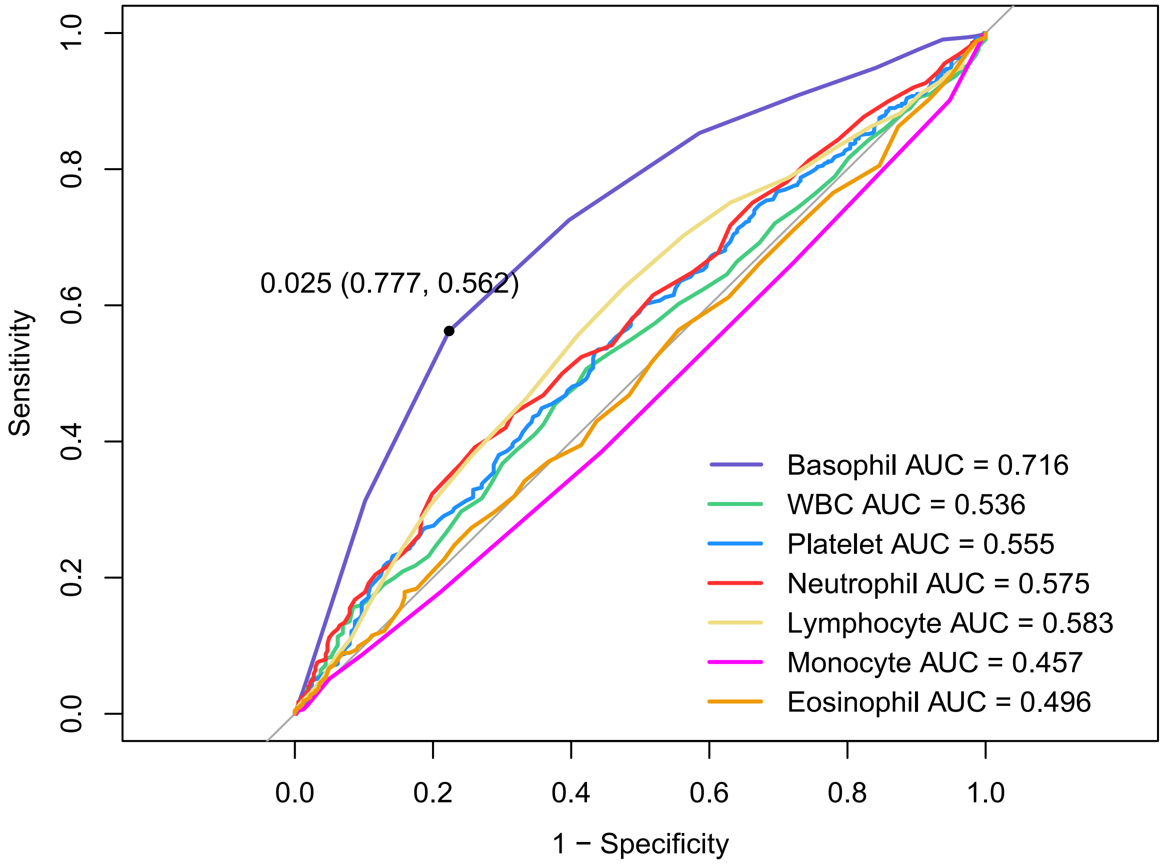

Supplement: Supplementary file 1 — Additional file 1: Figure S1. Receiver operating characteristic curves for immune/inflammation-relatedbiomarkers in blood routine examination, including basophil, white blood cell (WBC), platelet, neutrophil, lymphocyte, monocyte and eosinophil. [file 40169_2019_255_MOESM1_ESM.pdf]
