# Supplementary material for: Circulating basophil count as a prognostic marker of tumor aggressiveness and survival outcomes in colorectal cancer
Source: Clin Transl Med. 2020 Feb 10;9:6. doi: 10.1186/s40169-019-0255-4 (PMC7008108; doi:10.1186/s40169-019-0255-4)

Figure S2. X-tile analyses of DFS was performed to determine the optimal cut-off values for basophils count.

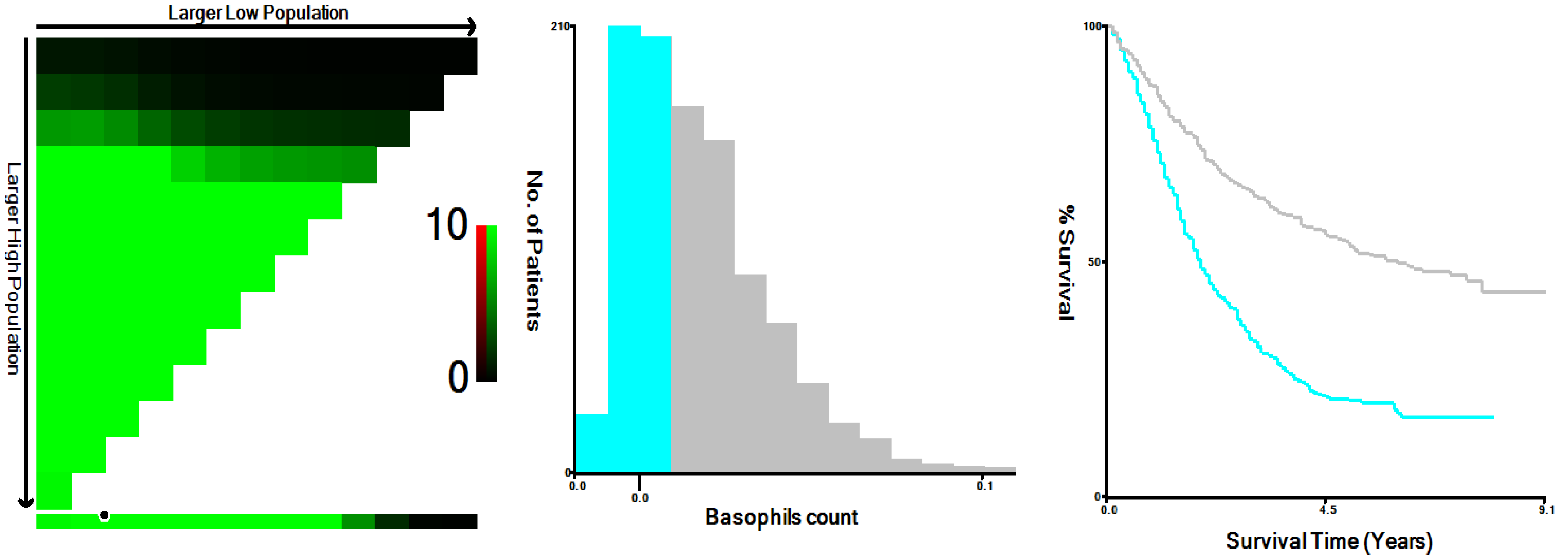

Supplement: Supplementary file 2 — Additional file 2: Figure S2. X-tile analyses of DFS were performed to determine the optimal cut-off valuesfor basophils count. [file 40169_2019_255_MOESM2_ESM.pdf]
