# Supplementary figures and images for: Circulating basophil count as a prognostic marker of tumor aggressiveness and survival outcomes in colorectal cancer
Source: Clin Transl Med. 2020 Feb 10;9:6. doi: 10.1186/s40169-019-0255-4 (PMC7008108; doi:10.1186/s40169-019-0255-4)

**A**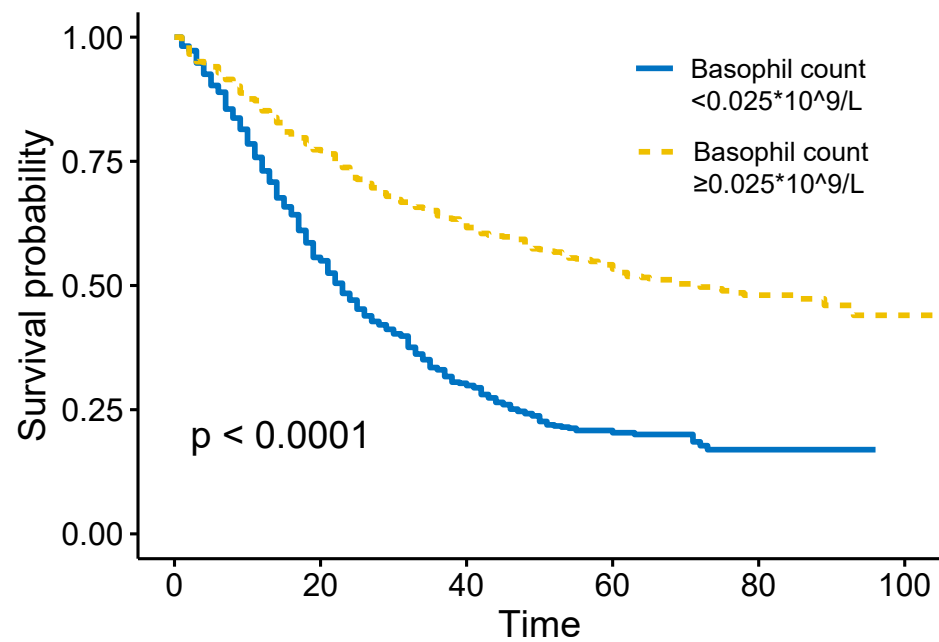**B**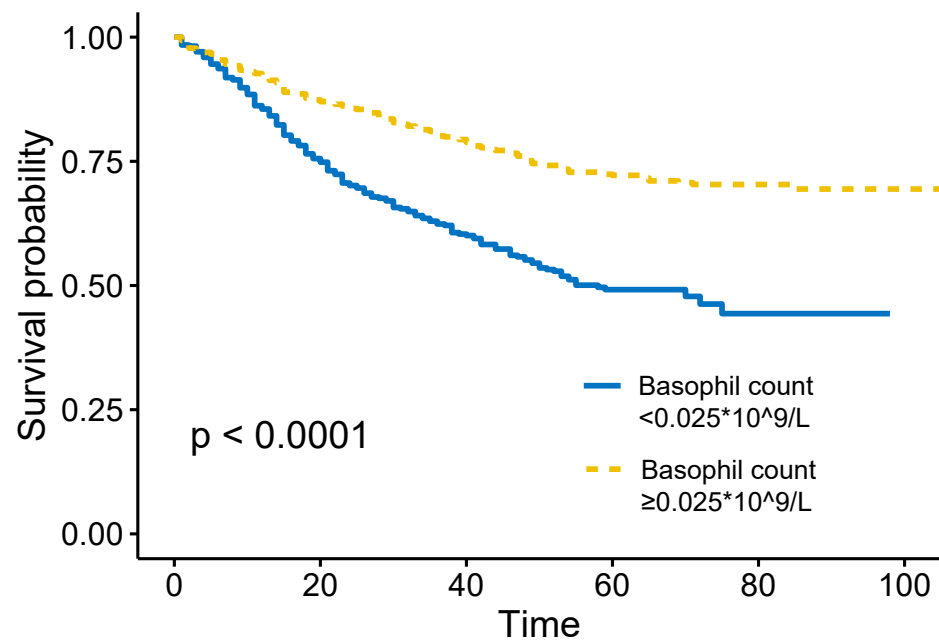

Supplement: Supplementary file 3 — Additional file 3: Figure S3. Kaplan–Meier (a). DFS and (b). OS curves of the serum basophil count level. [file 40169_2019_255_MOESM3_ESM.pdf]
