# Supplementary material for: Circulating basophil count as a prognostic marker of tumor aggressiveness and survival outcomes in colorectal cancer
Source: Clin Transl Med. 2020 Feb 10;9:6. doi: 10.1186/s40169-019-0255-4 (PMC7008108; doi:10.1186/s40169-019-0255-4)
Supplement: Supplementary file 4 — Additional file 4: Table S1. Comparison of baseline characteristics of the whole cohort by the serum basophil level. Table S2. OS of colorectal cancer after incorporation of serum basophil count level into conventional AJCC TNM staging system. [file 40169_2019_255_MOESM4_ESM.docx]

**Table S1. Comparison of baseline characteristics of the whole cohort by the serum basophil level.**

| **Variable** | **Serum basophil level (%)** | | ***P*** |
| --- | --- | --- | --- |
|  | **<0.025*10^9/L**  **(N=442)** | **≥0.025*10^9/L**  **(N=587)** |  |
| **T stage** |  |  | 0.024 |
| **T1** | 15 (3.4) | 28 (4.8) |  |
| **T2** | 56 (12.7) | 99 (16.9) |  |
| **T3** | 102 (23.1) | 143 (24.4) |  |
| **T4a** | 245 (55.4) | 303 (51.6) |  |
| **T4b** | 24 (5.4) | 14 (2.4) |  |
| **N stage** |  |  | 0.004 |
| **N0** | 151 (34.2) | 249 (42.4) |  |
| **N1a** | 34 (7.7) | 61 (10.4) |  |
| **N1b** | 50 (11.3) | 71 (12.1) |  |
| **N1c** | 73 (16.5) | 61 (10.4) |  |
| **N2a** | 64 (14.5) | 74 (12.6) |  |
| **N2b** | 70 (15.8) | 71 (12.1) |  |
| **Gender** |  |  | 0.824 |
| **Male** | 259 (59.6) | 348 (59.3) |  |
| **Female** | 183 (41.4) | 239 (40.7) |  |
| **Histology** |  |  | 0.121 |
| **Adenocarcinoma** | 359 (81.2) | 478 (81.4) |  |
| **Mucinous adenocarcinoma** | 69 (15.6) | 101 (17.2) |  |
| **Signet ring cell carcinoma** | 14 (3.2) | 8 (1.4) |  |
| **Tumor location** |  |  | 0.403 |
| **Right colon** | 116 (26.2) | 134 (22.8) |  |
| **Left colon** | 66 (14.9) | 98 (16.7) |  |
| **Rectum** | 260 (58.8) | 355 (60.5) |  |
| **Tumor grade** |  |  | 0.067 |
| **Well** | 23 (5.2) | 36 (6.1) |  |
| **Moderate** | 269 (60.9) | 391 (66.6) |  |
| **Poor** | 150 (33.9) | 160 (27.3) |  |
| **Venous invasion** |  |  | 0.031 |
| **No** | 279 (63.1) | 408 (69.5) |  |
| **Yes** | 163 (36.9) | 179 (30.5) |  |
| **Perineural invasion** |  |  | 0.009 |
| **No** | 298 (67.4) | 439 (74.8) |  |
| **Yes** | 144 (32.6) | 148 (25.2) |  |
| **Lymph nodes dissected in total** |  |  | <0.001 |
| **<12** | 62 (14.0) | 143 (24.4) |  |
| **≥12** | 380 (86.0) | 444 (75.6) |  |
| **Age (years)** |  |  | 0.032 |
| **<65** | 287 (64.9) | 418 (71.2) |  |
| **≥65** | 155 (35.1) | 169 (28.8) |  |
| **Adjuvant chemotherapy** |  |  | 0.179 |
| **No** | 89 (20.1) | 99 (16.9) |  |
| **Yes** | 353 (79.9) | 488 (83.1) |  |
| **Serum CEA level** |  |  | <0.001 |
| **Normal** | 229 (51.8) | 369 (62.9) |  |
| **Elevated** | 213 (48.2) | 218 (37.1) |  |

**Table S2. OS of colorectal cancer after incorporation of serum basophil count level into conventional AJCC TNM staging system**

| **AJCC TNM staging system** | | | |  | **After incorporation of C-stage into AJCC stage** | | | |
| --- | --- | --- | --- | --- | --- | --- | --- | --- |
| **Stage** | **No. of patients** | **Overall survival** | |  | **Stage** | **No. of patients** | **Overall survival** | |
|  |  | **HR (95% CI)** | **P value** |  |  |  | **HR (95% CI)** | **P value** |
| **Ⅰ** | 141 | 1 | \ |  | **Ⅰ B-** | 49 | 1 | \ |
|  |  |  |  |  | **Ⅰ B+** | 92 | 0.460 (0.177-1.196) | 0.111 |
| **ⅡA** | 123 | 1.945 (1.053-3.592) |  |  | **ⅡA B-** | 50 | 1.769 (0.779-4.016) | 0.173 |
|  |  |  |  |  | **ⅡA B+** | 73 | 1.001 (0.417-2.398) | 0.999 |
| **ⅡB** | 128 | 1.483 (0.777-2.829) |  |  | **ⅡB B-** | 46 | 1.543 (0.645-3.694) | 0.330 |
|  |  |  |  |  | **ⅡB B+** | 82 | 0.659 (0.265-1.640) | 0.370 |
| **ⅡC** | 8 | 2.188 (0.627-7.633) |  |  | **ⅡC B-** | 6 | 2.813 (0.744-10.639) | 0.128 |
|  |  |  |  |  | **ⅡC B+** | 2 | NA | NA |
| **ⅢA** | 44 | 1.927 (0.785-4.732) |  |  | **ⅢA B-** | 16 | 1.920 (0.580-6.353) | 0.285 |
|  |  |  |  |  | **ⅢA B+** | 28 | 0.876 (0.235-3.270) | 0.844 |
| **ⅢB** | 327 | 3.733 (2.158-6.460) |  |  | **ⅢB B-** | 148 | 3.159 (1.532-6.511) | 0.002 |
|  |  |  |  |  | **ⅢB B+** | 179 | 2.091 (1.005-4.349) | 0.048 |
| **ⅢC** | 258 | 6.728 (3.866-11.708) |  |  | **ⅢC B-** | 127 | 6.283 (3.050-12.943) | <0.001 |
|  |  |  |  |  | **ⅢC B+** | 131 | 3.362 (1.619-6.980) | 0.001 |
